# Data regarding the influence of Al, Ti, and C additions to as-cast Al_0.6_CoCrFeNi compositionally complex alloys on microstructures and mechanical properties

**DOI:** 10.1016/j.dib.2019.104742

**Published:** 2019-11-04

**Authors:** Alex Asabre, Janine Pfetzing-Micklich, Oleg Stryzhyboroda, Aleksander Kostka, Ulrike Hecht, Guillaume Laplanche

**Affiliations:** a Institut für Werkstoffe, Ruhr-Universität Bochum, Universitätsstr. 150, 44801 Bochum, Germany; b Zentrum für Grenzflächendominierte Höchstleistungswerkstoffe (ZGH), Ruhr-University Bochum, Universitätsstr. 150, 44801 Bochum, Germany; c ACCESS e.V., Intzestraße 5, D-52072 Aachen, Germany

**Keywords:** High-entropy alloys, HEA, CCA, X-ray diffraction, Tensile properties, Widmanstätten microstructures, Microstructural refinement

## Abstract

This brief paper contains raw data of X-ray diffraction (XRD) measurements, microstructural characterization, chemical compositions, and mechanical properties describing the influence of Al, Ti, and C on as-cast Al_0.6_CoCrFeNi compositionally complex alloys (CCAs). The presented data are related to the research article in reference [1] and therefore this article can be referred to as for the interpretation of the data. X-ray diffraction data presented in this paper are measurements of 2*θ* versus intensities for each studied alloy. A Table lists the obtained lattice parameters of each identified phase determined by Rietveld analysis. Microstructural-characterization data reported here include backscattered electron (BSE) micrographs taken at different magnifications in a scanning electron microscope (SEM) of Widmanstätten and dendritic microstructures and microstructural parameters such as phase volume fractions, thickness of face-centered cubic (FCC) plates, and prior grain sizes. The compositions of the identified individual phases determined by energy-dispersive X-ray spectroscopy (EDX) in the transmission electron microscope (TEM) are listed as well. Finally, mechanical data including engineering stress-strain curves obtained at different temperatures (room temperature, 400 °C, and 700 °C) for all CCAs are reported.

**Table:** Specifications Table

Subject	Metals and Alloys
Specific subject area	Alloy Design of compositionally complex alloys (CCAs)
Type of data	Tables Images (SEM-BSE) Excel files (XRD, stress-strain data)
How data were acquired	XRD: PANalytical X'Pert Pro MRD diffractometer, SEM: Quanta FEI 650 ESEM, TEM-EDX: FEI Tecnai F20 G^2^ S-Twin ImageJ: Image Analysis Software, Tensile testing machine: Zwick Roell XForce Z100
Data format	Measured raw (XRD patterns, stress-strain curves), Analyzed (prior grain sizes, thickness of FCC plates and volume fraction of FCC phase)
Parameters for data collection	XRD patterns acquired using a PANalytical X'Pert Pro MRD diffractometer operating with a Cu-Kα radiation source (λ = 1.54 Å). BSE images were obtained using an SEM of type Quanta FEI 650 ESEM with an acceleration voltage of 20 kV and a working distance of 10 mm. Tensile tests were performed at three different temperatures using a Zwick Roell Z100 Xforce machine at a strain rate of 10^−3^ s^−1^.
Description of data collection	Metallographic samples were prepared by grinding and polishing. These samples were used for collecting the XRD and SEM data. The chemical composition data were measured in the TEM from prepared TEM foils. Additionally the tensile data were acquired by tensile testing cylindrical dog-bone-shaped specimens.
Data source location	Institution: Institut für Werkstoffe, Ruhr-Universität Bochum, City/Region: Bochum/North Rhine-Westphalia (NRW), Country: Germany
Data accessibility	Data are hosted with the article
Related research article	**Authors name**: A. Asabre, A. Kostka, O. Stryzhyboroda, J. Pfetzing-Micklich, U. Hecht, G. Laplanche **Title**: Effect of Al, Ti and C additions on Widmanstätten microstructure and mechanical properties of cast Al_0.6_CoCrFeNi compositionally complex alloys Journal: Materials & Design **DOI**: 10.1016/j.matdes.2019.108201

**Table:** 

**Value of the Data** • The presented data constitute basic data regarding the effect of micro-alloying of Al, Ti, and C on phase stability, microstructural and mechanical properties of an Al_0.6_CoCrFeNi CCA. • These datasets facilitate open access and reproducibility of buried information in the related article. • All micrographs and numerical datasets here could be used to promote the development of new algorithms for image analysis for fast and efficient quantitative microstructural analysis. • The alloy chemistry, the compositions of the individual phases present in the alloys as well as their mechanical and microstructural properties could be implemented in programs used for alloy design.

## 1 Data

The XRD data present in this paper are 2*θ* versus intensity data obtained from our diffractometer for all investigated alloys. The XRD data are provided as Excel files (see Zip file) and a summary of the plotted diffraction patterns is shown in Fig. B2 of supplementary material of Ref. [1]. The calculated lattice parameters together with their standard deviation for each detected phase are listed in Table 1 for all investigated alloys. BSE micrographs taken at different magnifications are presented to document the effect of micro-alloying on microstructure (see Zip file). For all CCAs, five BSE micrographs are provided, one low-magnification image with a remnant indent spanning all phases present in the alloy and four other high-magnification micrographs. From some of these BSE images, the microstructural parameters, i.e. mean prior grain size (*d*) and average thickness of FCC plates (*λ*) listed in Table 2, were determined. In addition, the surface area fractions of TiC-particles listed in Table 3 were obtained from BSE images using an image analysis software (ImageJ). The local chemical compositions of the phases measured at three different locations in a transmission electron microscope for the Ti_3_C_0.25_ CCA are given in Table 4. The mean volume fraction of the FCC phase provided in Table 2 were determined based on these TEM-EDX measurements in combination with a mass balance, see section D of supplementary material of Ref. [1]. The raw tensile data obtained at different temperatures are provided as Excel files (see supplementary Zip file). These Excel files are labeled as follows: alloy name, temperature at which the test was performed, test number (for reproducibility) and whether this test was presented in a figure of the related article [1]. For example, “Tensile Test_Al_13_-400°C-1st_selected” means that a “Tensile test” was performed for the alloy named “Al_13_” at a temperature of “400 °C”. “1st” means that this dataset corresponds to the first tensile test performed for this alloy and “selected” indicates that this data was presented in the related article [1]. Note that even though only one test per alloy and temperature was presented in the related paper [1], both tests performed for each alloy and temperature, i.e. “selected” or “Not selected” tests were considered to assess the reproducibility of the tensile tests. In each Excel sheet, the name of the alloy is provided together with the height, gauge diameter, and gauge length of the tensile specimen. The time, force acting on the specimen, and cross-head displacement are given in the first three columns of the Excel sheets while engineering stress, engineering strain, and engineering plastic strain are provided in the fourth, fifth, and sixth columns, respectively.

**Table 1:** Lattice parameters and standard deviations of the identified face-centered cubic (FCC) and body-centered cubic (BCC) phases for all investigated drop cast CCAs.

Names	Nominal composition in at.%	*a*_FCC_ nm	Stdev (*a*_FCC_) nm	*a*_BCC_ nm	Stdev (*a*_BCC_) nm
Al_13_	Al_13.00_Co_21.74_Cr_21.74_Fe_21.74_Ni_21.74_	0.3602	2E-4	0.2884	2E-4
C_0.25_	Al_13.00_Co_21.69_Cr_21.69_Fe_21.69_Ni_21.69_C_0.25_	0.3616	2E-4	0.2886	2E-4
Al_16_	Al_16.00_Co_21.00_Cr_21.00_Fe_21.00_Ni_21.00_	0.3601	2E-4	0.2879	2E-4
Ti_3_	Al_13.00_Co_21.00_Cr_21.00_Fe_21.00_Ni_21.00_Ti_3.00_	0.3605	2E-4	0.2889	2E-4
Ti_3_C_0.25_	Al_13.00_Co_20.94_Cr_20.94_Fe_20.94_Ni_20.94_Ti_3.00_C_0.25_	0.3603	2E-4	0.2890	2E-4

**Table 2:** Microstructure parameters: mean prior grain size (*d*), average FCC volume fraction and mean thickness of FCC plate (*λ*) of all studied alloys. Mean deviation for these parameters = ± 5%.

Names	Nominal composition in at.%	Prior grain size, *d* (μm)	FCC vol. fraction (%)	FCC plate thickness, *λ* (μm)
Al_13_	Al_13.00_Co_21.74_Cr_21.74_Fe_21.74_Ni_21.74_	108	65	1.33
C_0.25_	Al_13.00_Co_21.69_Cr_21.69_Fe_21.69_Ni_21.69_C_0.25_	–	82	14.00
Al_16_	Al_16.00_Co_21.00_Cr_21.00_Fe_21.00_Ni_21.00_	>300	45	0.39
Ti_3_	Al_13.00_Co_21.00_Cr_21.00_Fe_21.00_Ni_21.00_Ti_3.00_	55	55	0.47
Ti_3_C_0.25_	Al_13.00_Co_20.94_Cr_20.94_Fe_20.94_Ni_20.94_Ti_3.00_C_0.25_	42	51	0.43

**Table 3:** Surface area fractions of TiC-particles in the CCAs with Ti + C additions.

Names	TiC surface area fraction %
Al_13_	–
Ti_3_C_0.25_	0.5
Ti_3_C_0.5_	1.25

**Table 4:** Raw chemical compositions in at.% of the phases (FCC, BCC and ordered BCC (B2)) present in the Ti_3_C_0.25_ CCA measured by EDX point analysis in the TEM. The absolute experimental error is ±2% except for Ti.

Phase	Al	Co	Cr	Fe	Ni	Ti
FCC	5.56	22.82	24.24	25.16	20.89	1.33
FCC	6.55	20.91	24.76	25.84	20.13	1.81
FCC	6.15	21.96	26.50	25.71	18.57	1.11
**mean**	**6.09**	**21.90**	**25.17**	**25.57**	**19.86**	**1.42**
BCC	1.56	17.89	46.85	28.06	5.36	0.28
BCC	1.76	15.77	47.56	26.45	7.84	0.62
BCC	4.03	17.45	43.80	25.52	8.32	0.88
**mean**	**2.45**	**17.04**	**46.07**	**26.68**	**7.17**	**0.59**
B2	25.16	20.81	5.95	11.32	29.98	6.78
B2	21.53	20.23	9.74	12.07	29.81	6.62
B2	23.19	20.24	8.73	12.26	28.37	7.21
**mean**	**23.29**	**20.43**	**8.14**	**11.88**	**29.39**	**6.87**

## 2 Experimental design, materials, and methods

XRD diffraction pattern for all investigated CCAs were acquired using a diffractometer of type PANalytical X'Pert Pro MRD operating with a Cu-Kα radiation source (λ = 1.54 Å). The diffraction patterns were measured within a 2*θ*-range of 20° and 120°, a step size of 0.006° and an integration time of 280 s. The lattice parameter of the indexed phases listed in Table 1 were determined by Rietveld analysis using the MAUD program. The backscattered electron (BSE) images were taken using a Quanta FEI 650 scanning electron microscope (SEM) with an accelerating voltage of 20 kV and a working distance between ∼5 and 10 mm, for more details see Ref. [2]. The mean prior grain size (*d*) in Table 2 was determined using the Heyn linear intercept method outlined in ASTM E−112, see Ref. [3] for a detailed description of its application. The mean thickness of the FCC plates (*λ*) was measured using Imagic ims client software. In this software, several lines were drawn perpendicular to the plates to determine its average thickness. The average volume fraction of individual phases listed in Table 2 was determined using a mass balance presented in section D of the supplementary material of Ref. [1]. The evolution of TiC-particles in the CCAs listed in Table 3 were obtained using an image analysis software “ImageJ 1.51k”. The ImageJ software was used to collect binary images from which the surface area fractions were determined, for more details see Ref. [4]. The average chemical compositions of the phases given in Table 4 were determined using energy dispersive spectroscopy (EDX) in an FEI Tecnai F20 G^2^ S-Twin TEM.

Tensile tests were performed in a Zwick Roell XForce Z100 machine at three different temperatures: room temperature (RT), 400 °C, and 700 °C using a strain rate of 10^−3^ s^−1^, for more details see Ref. [5].

## References

[1] Asabre A., Kostka A., Stryzhyboroda O., Pfetzing-Micklich J., Hecht U., Laplanche G. (2019). Effect of Al, Ti and C additions on microstructural refinement and mechanical properties of cast Al_0.6_CoCrFeNi compositionally complex alloy with Widmanstätten microstructures. Mater. Des..

[2] Laplanche G., Schneider M., Scholz F., Frenzel J., Eggeler G., Schreuer J. (2020). Processing of a single-crystalline CrCoNi medium-entropy alloy and evolution of its thermal expansion and elastic stiffness coefficients with temperature. Scr. Mater..

[3] Schneider M., George E.P., Manescau T.J., Záležák T., Hunfeld J., Dlouhý A., Eggeler G., Laplanche G. (2019). Analysis of strengthening due to grain boundaries and annealing twin boundaries in the CrCoNi medium-entropy alloy. Int. J. Plast..

[4] Laplanche G., Berglund S., Reinhart C., Kostka A., Fox F., George E.P. (2018). Phase stability and kinetics of σ-phase precipitation in CrMnFeCoNi high-entropy alloys. Acta Mater..

[5] Schneider M., Werner F., Langenkämper D., Reinhart C., Laplanche G. (2019). Effect of temperature and texture on Hall–Petch strengthening by grain and annealing twin boundaries in the MnFeNi medium-entropy alloy. Metals.

