# Supplementary figures and images for: Data regarding the influence of Al, Ti, and C additions to as-cast Al0.6CoCrFeNi compositionally complex alloys on microstructures and mechanical properties
Source: Data Brief. 2019 Nov 4;27:104742. doi: 10.1016/j.dib.2019.104742 (PMC6864337; doi:10.1016/j.dib.2019.104742)

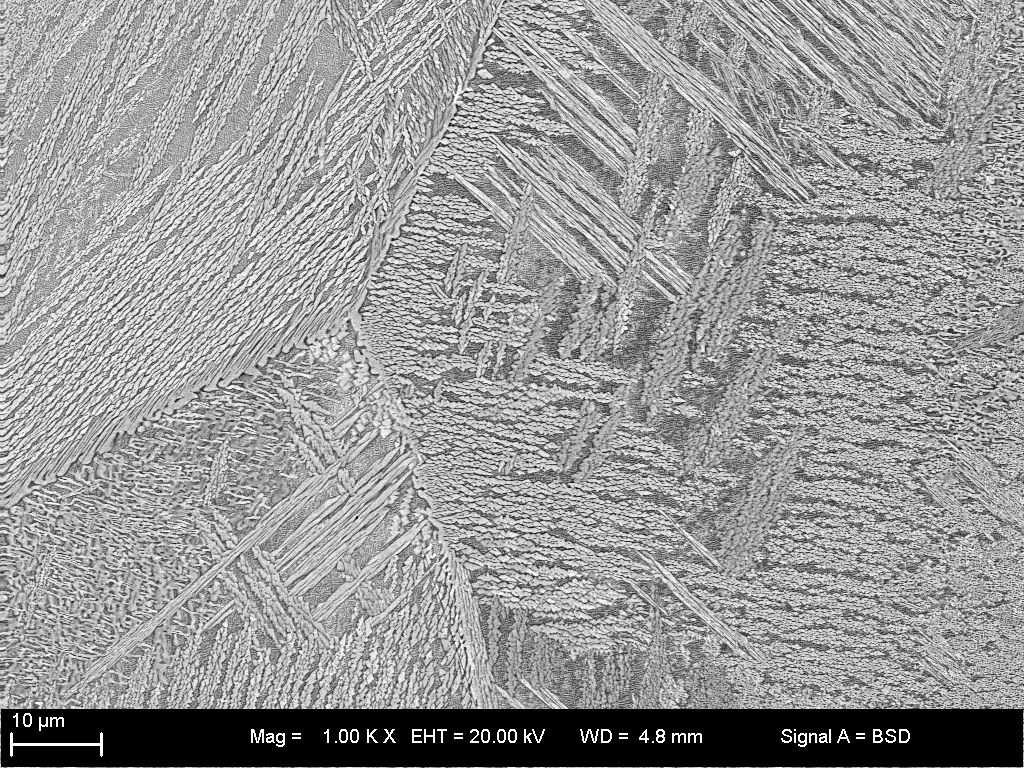

Supplement: Multimedia component 1 [file mmc1.zip › data in brief_supplementary material zip/BSE-Images_Microstructure evolution/Al13/Al13_As cast_1000x_ (2).tif]

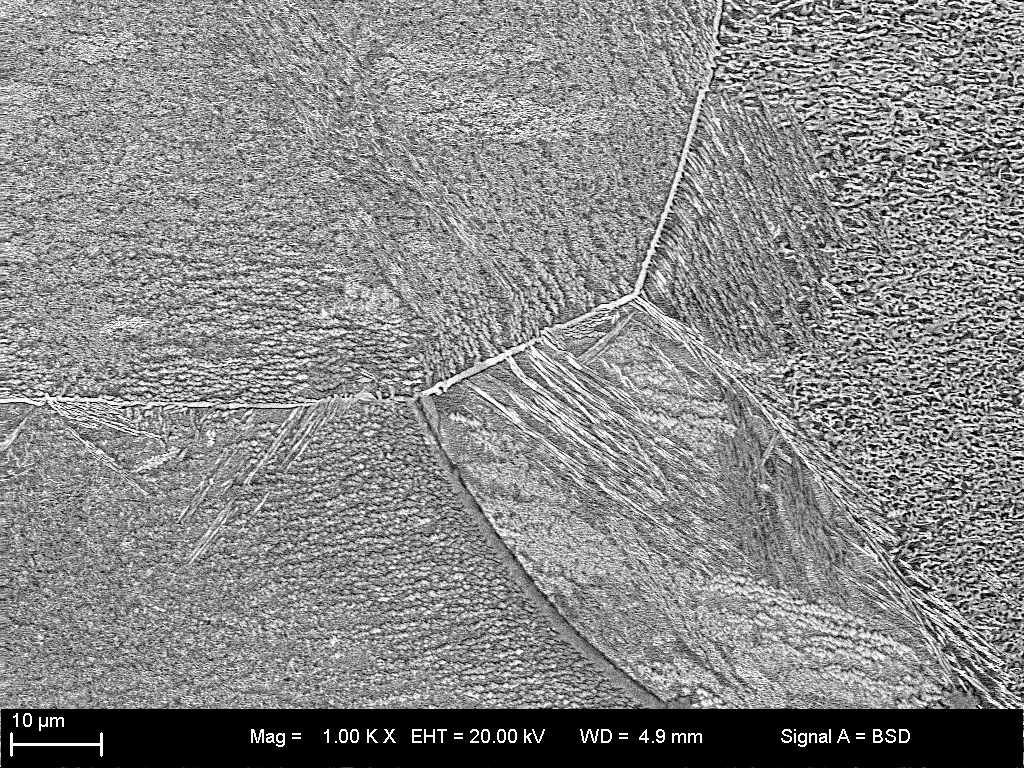

Supplement: Multimedia component 1 [file mmc1.zip › data in brief_supplementary material zip/BSE-Images_Microstructure evolution/Al13/Al13_As cast_1000x_.tif]

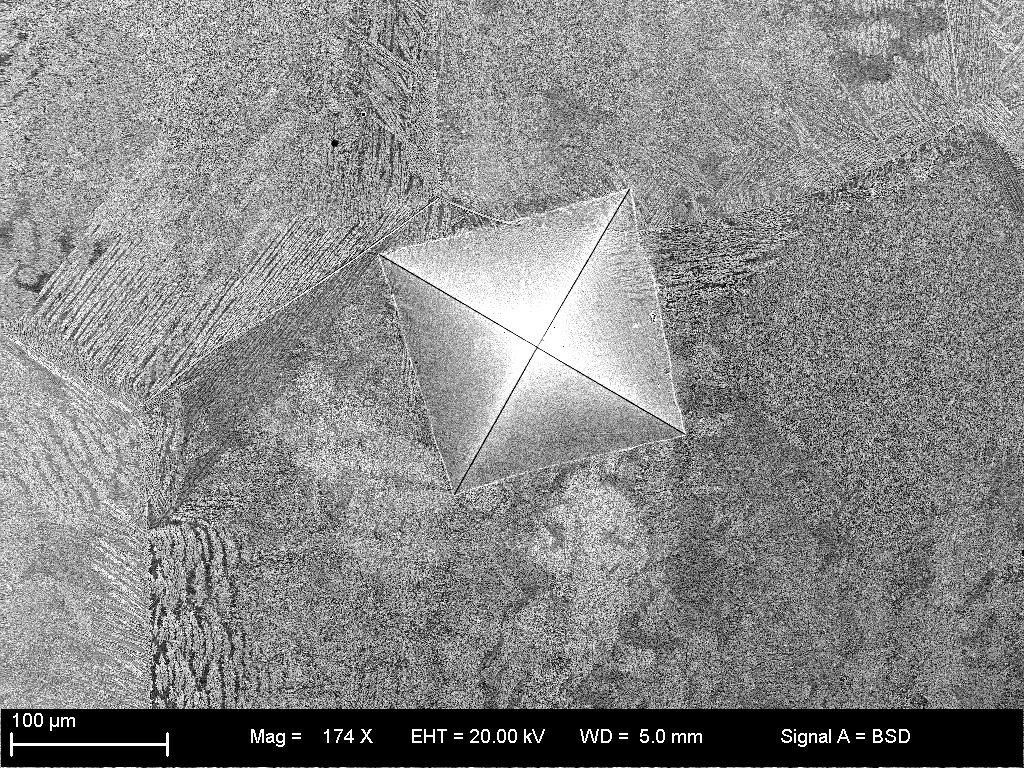

Supplement: Multimedia component 1 [file mmc1.zip › data in brief_supplementary material zip/BSE-Images_Microstructure evolution/Al13/Al13_As cast_174x_.tif]

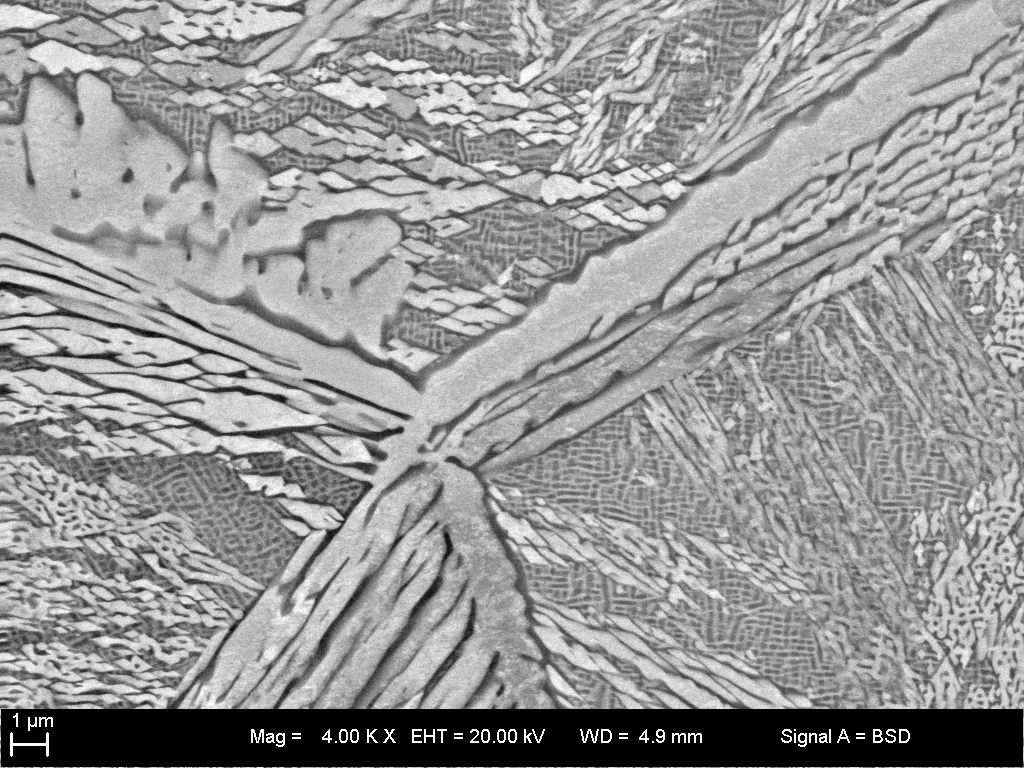

Supplement: Multimedia component 1 [file mmc1.zip › data in brief_supplementary material zip/BSE-Images_Microstructure evolution/Al13/Al13_As cast_4000x_ (2).tif]

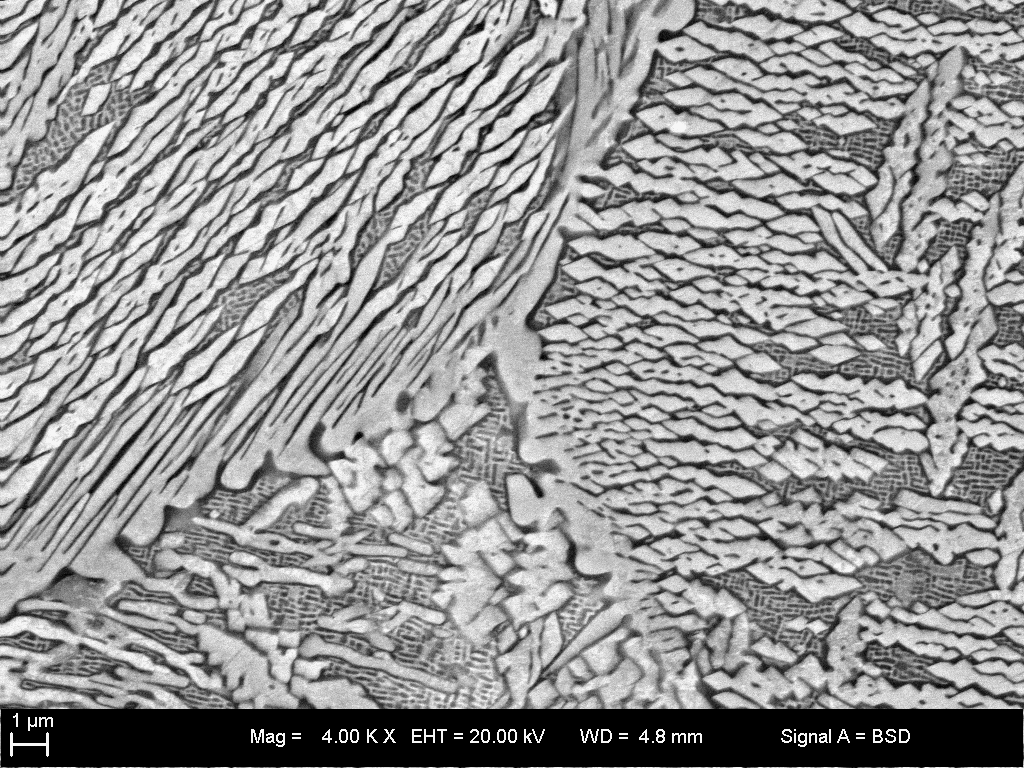

Supplement: Multimedia component 1 [file mmc1.zip › data in brief_supplementary material zip/BSE-Images_Microstructure evolution/Al13/Al13_As cast_4000x_.tif]

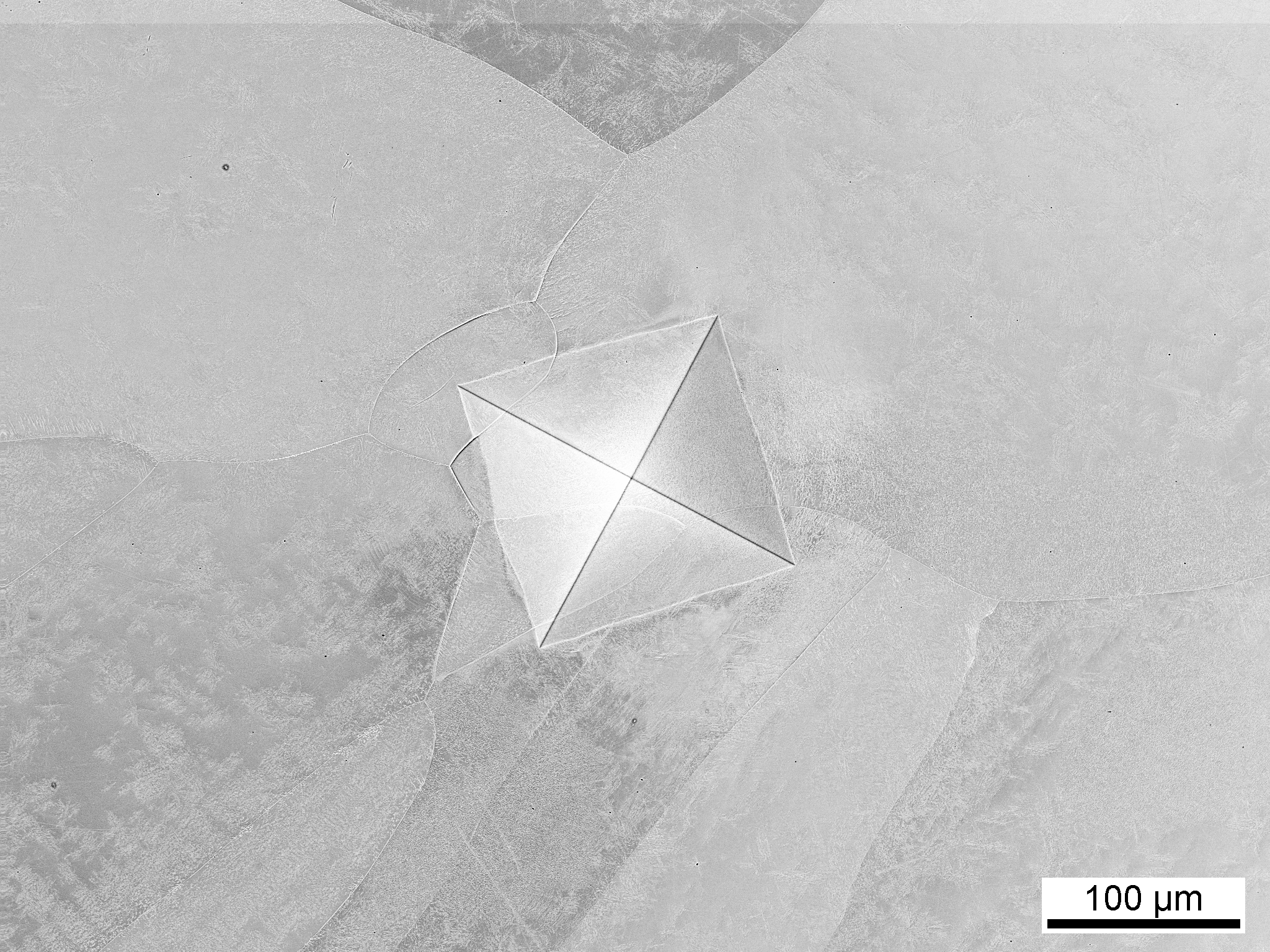

Supplement: Multimedia component 1 [file mmc1.zip › data in brief_supplementary material zip/BSE-Images_Microstructure evolution/Al16/Al16_As cast_174x_.tif]

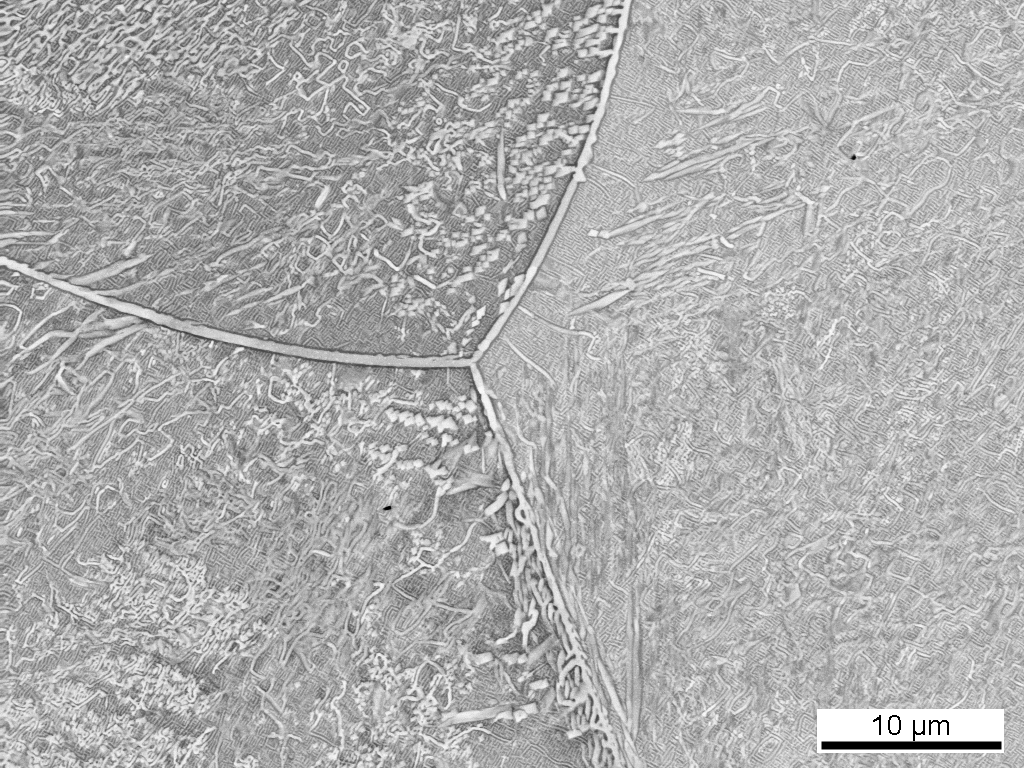

Supplement: Multimedia component 1 [file mmc1.zip › data in brief_supplementary material zip/BSE-Images_Microstructure evolution/Al16/Al16_As cast_2000x_.tiff]

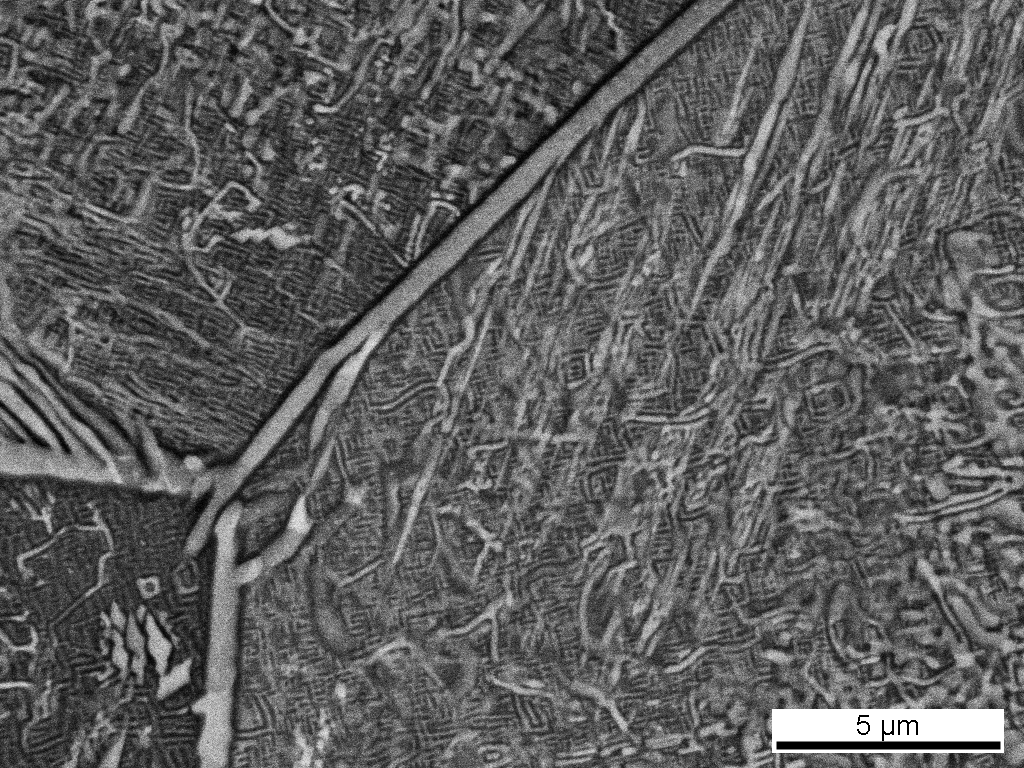

Supplement: Multimedia component 1 [file mmc1.zip › data in brief_supplementary material zip/BSE-Images_Microstructure evolution/Al16/Al16_As cast_3000x_.tif]

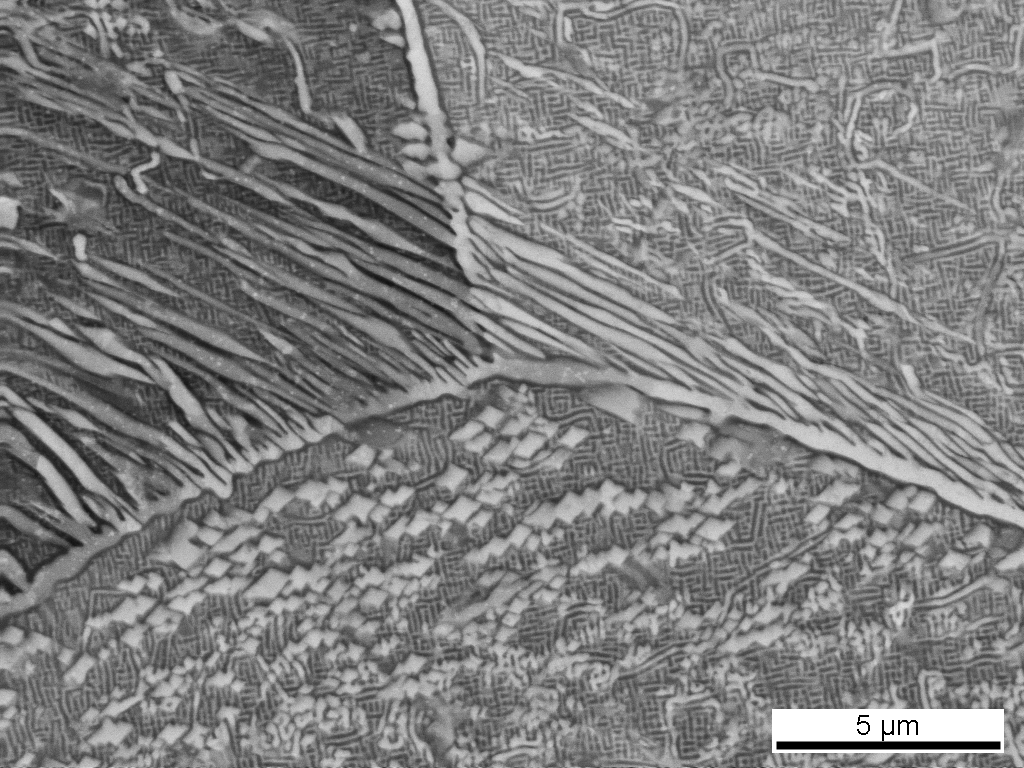

Supplement: Multimedia component 1 [file mmc1.zip › data in brief_supplementary material zip/BSE-Images_Microstructure evolution/Al16/Al16_As cast_5000x_.tiff]

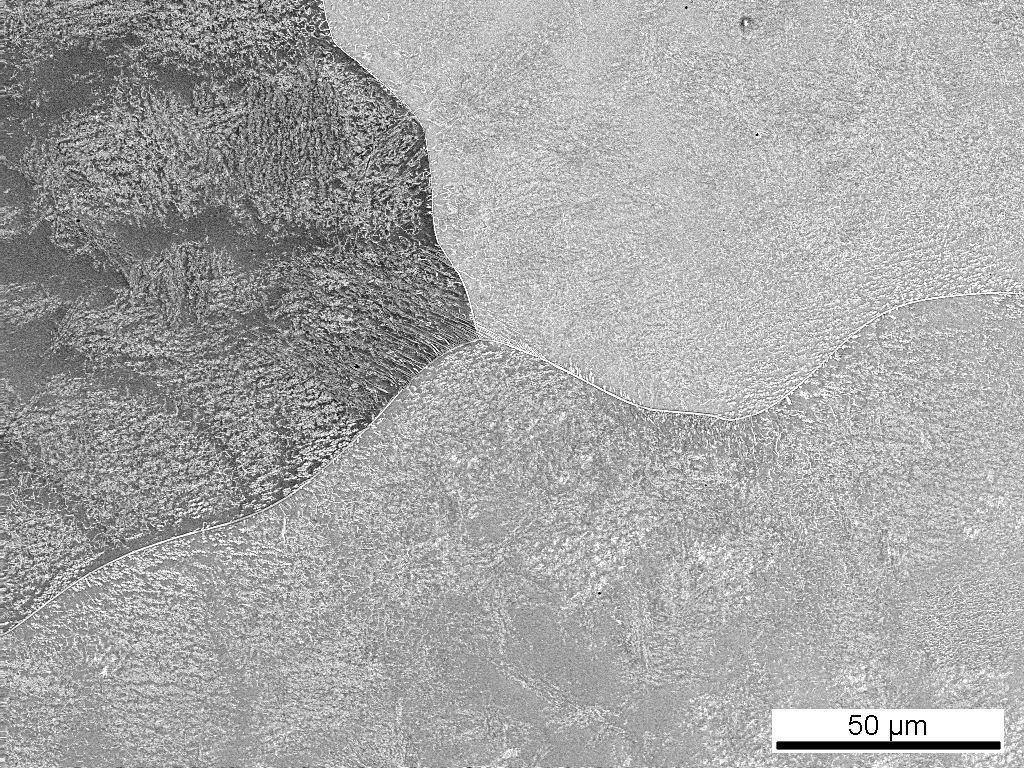

Supplement: Multimedia component 1 [file mmc1.zip › data in brief_supplementary material zip/BSE-Images_Microstructure evolution/Al16/Al16_As cast_500x_.tif]

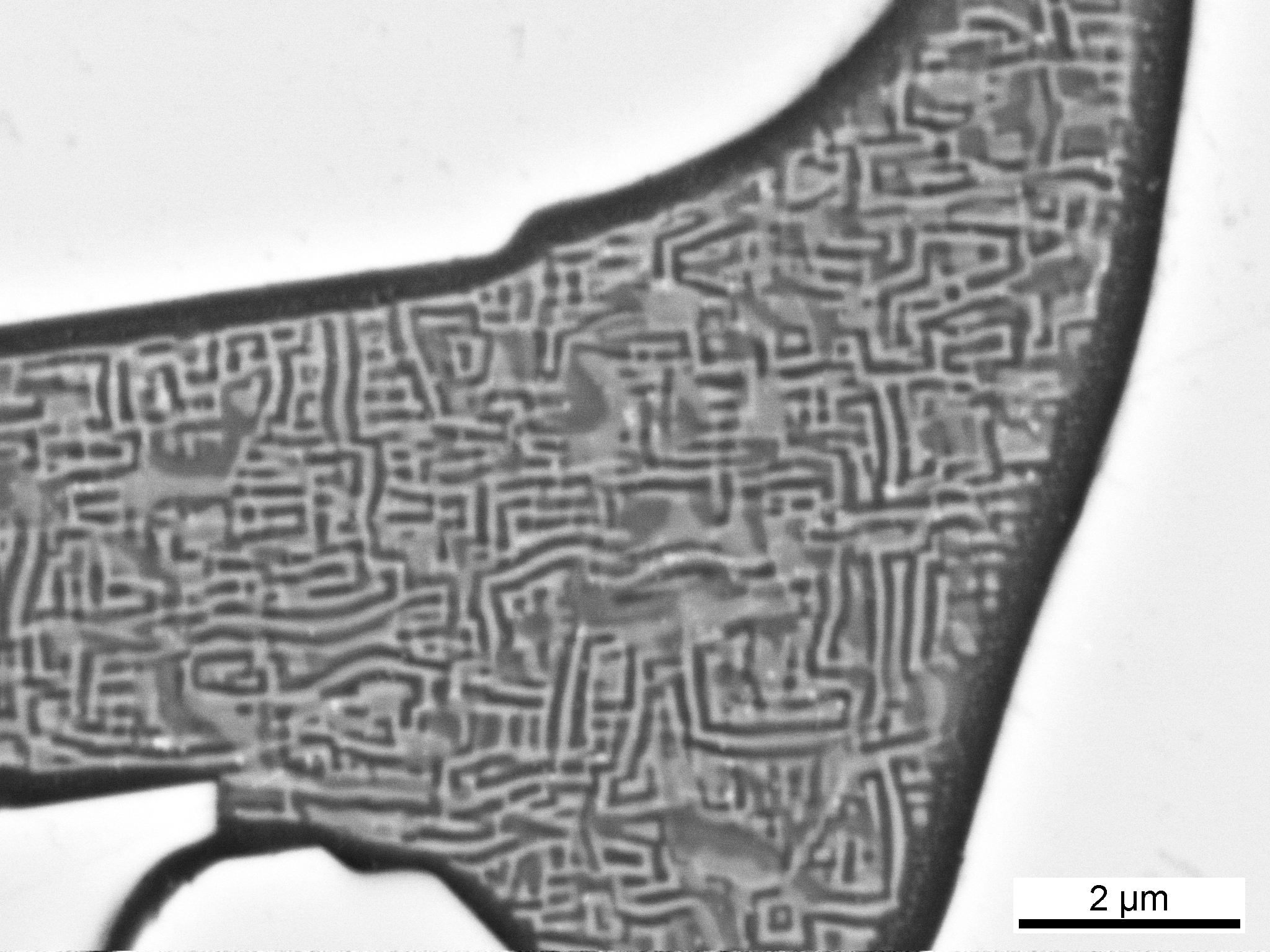

Supplement: Multimedia component 1 [file mmc1.zip › data in brief_supplementary material zip/BSE-Images_Microstructure evolution/C0.25/C0,25_As cast_10000x_.tif]

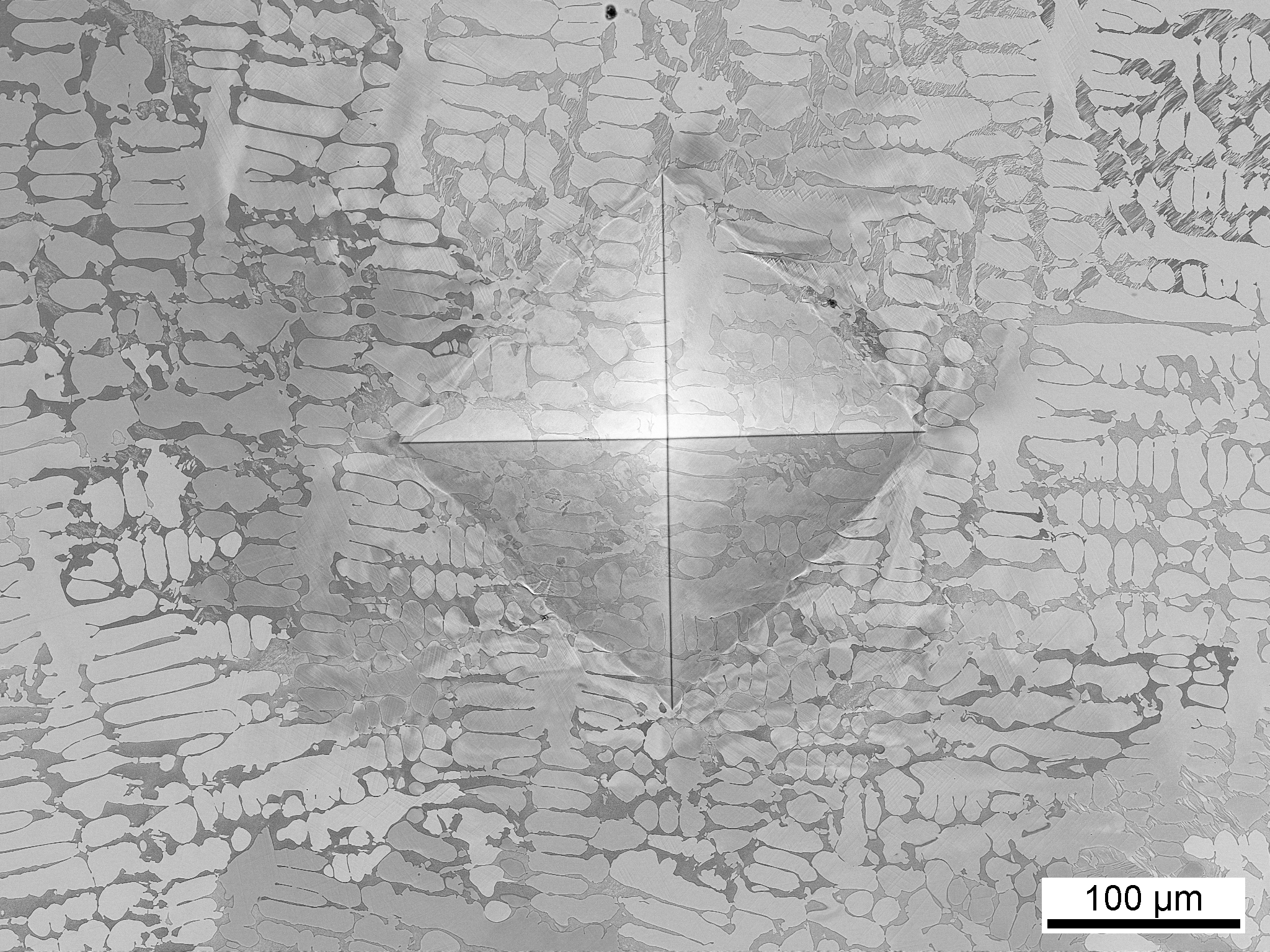

Supplement: Multimedia component 1 [file mmc1.zip › data in brief_supplementary material zip/BSE-Images_Microstructure evolution/C0.25/C0,25_As cast_174x_.tif]

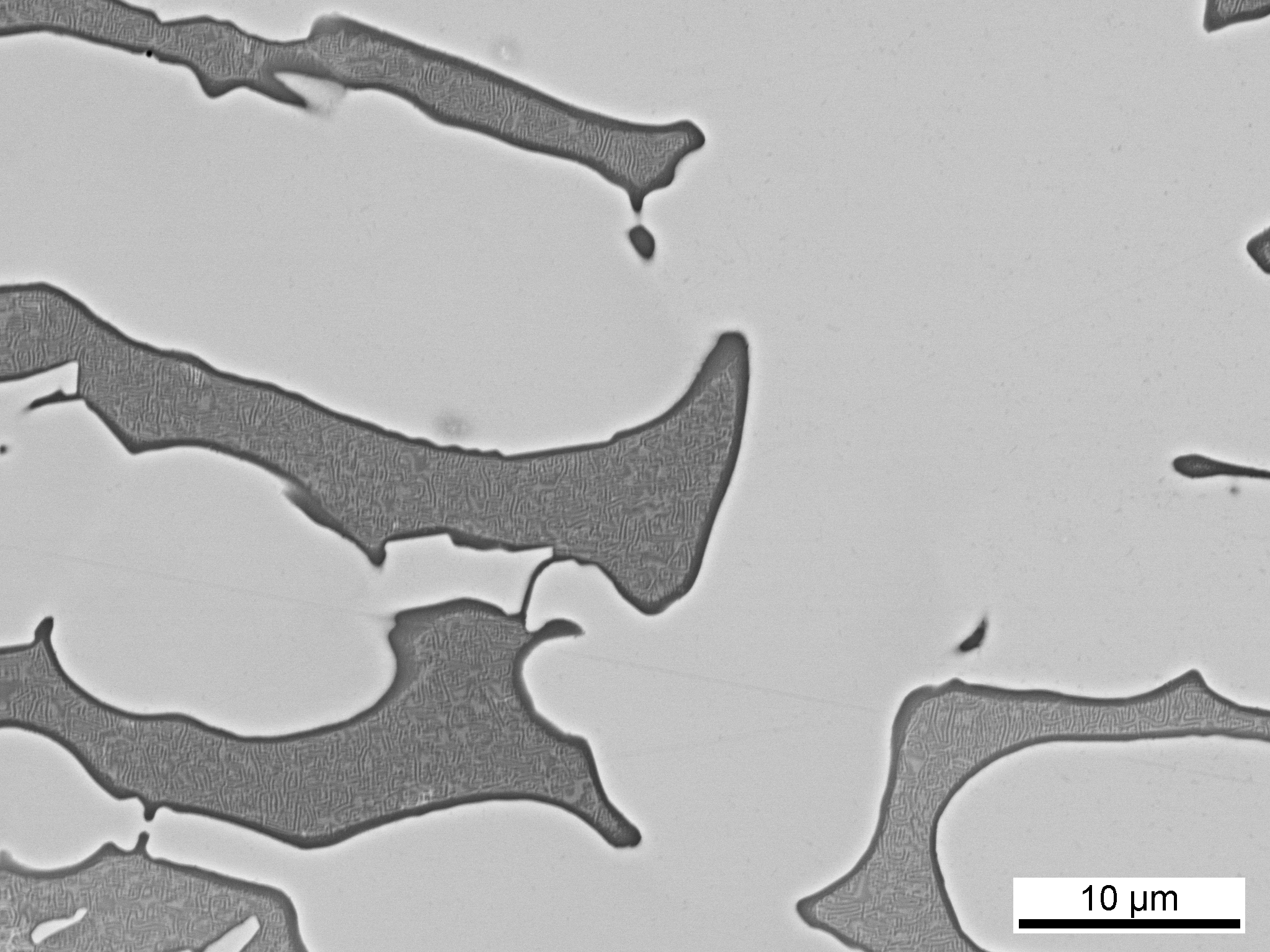

Supplement: Multimedia component 1 [file mmc1.zip › data in brief_supplementary material zip/BSE-Images_Microstructure evolution/C0.25/C0,25_As cast_2000x_.tif]

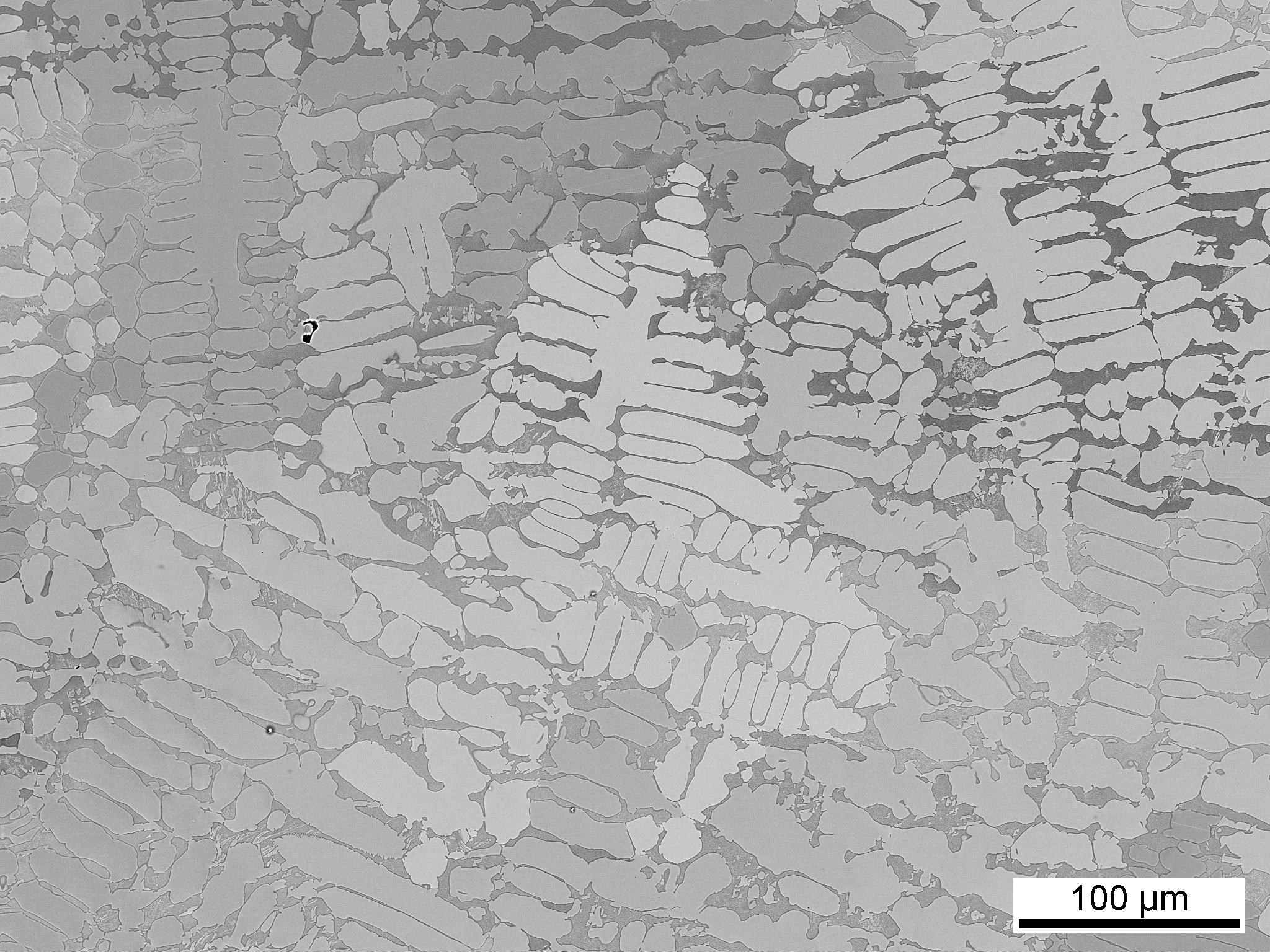

Supplement: Multimedia component 1 [file mmc1.zip › data in brief_supplementary material zip/BSE-Images_Microstructure evolution/C0.25/C0,25_As cast_200x_.tif]

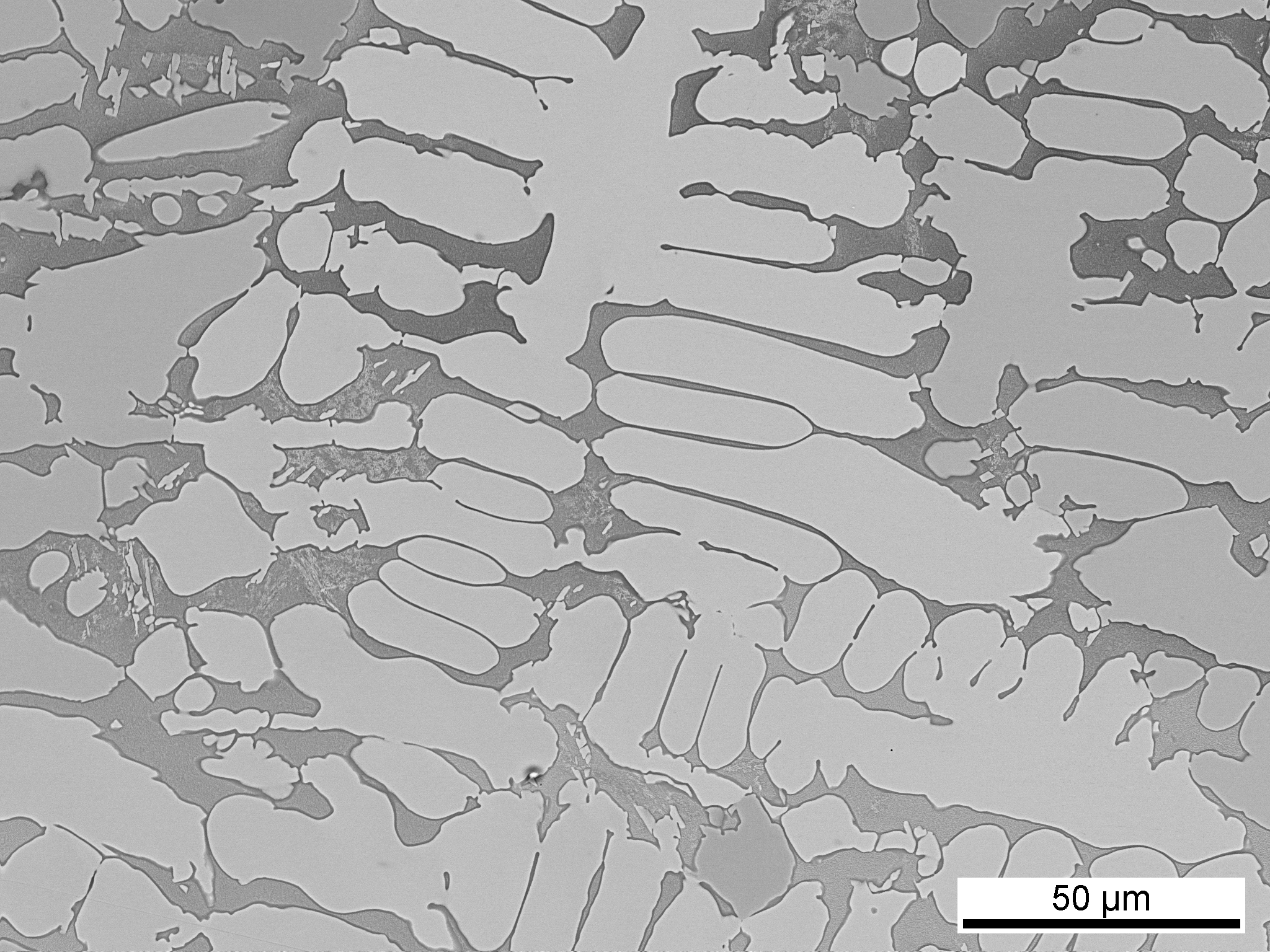

Supplement: Multimedia component 1 [file mmc1.zip › data in brief_supplementary material zip/BSE-Images_Microstructure evolution/C0.25/C0,25_As cast_500x_.tif]

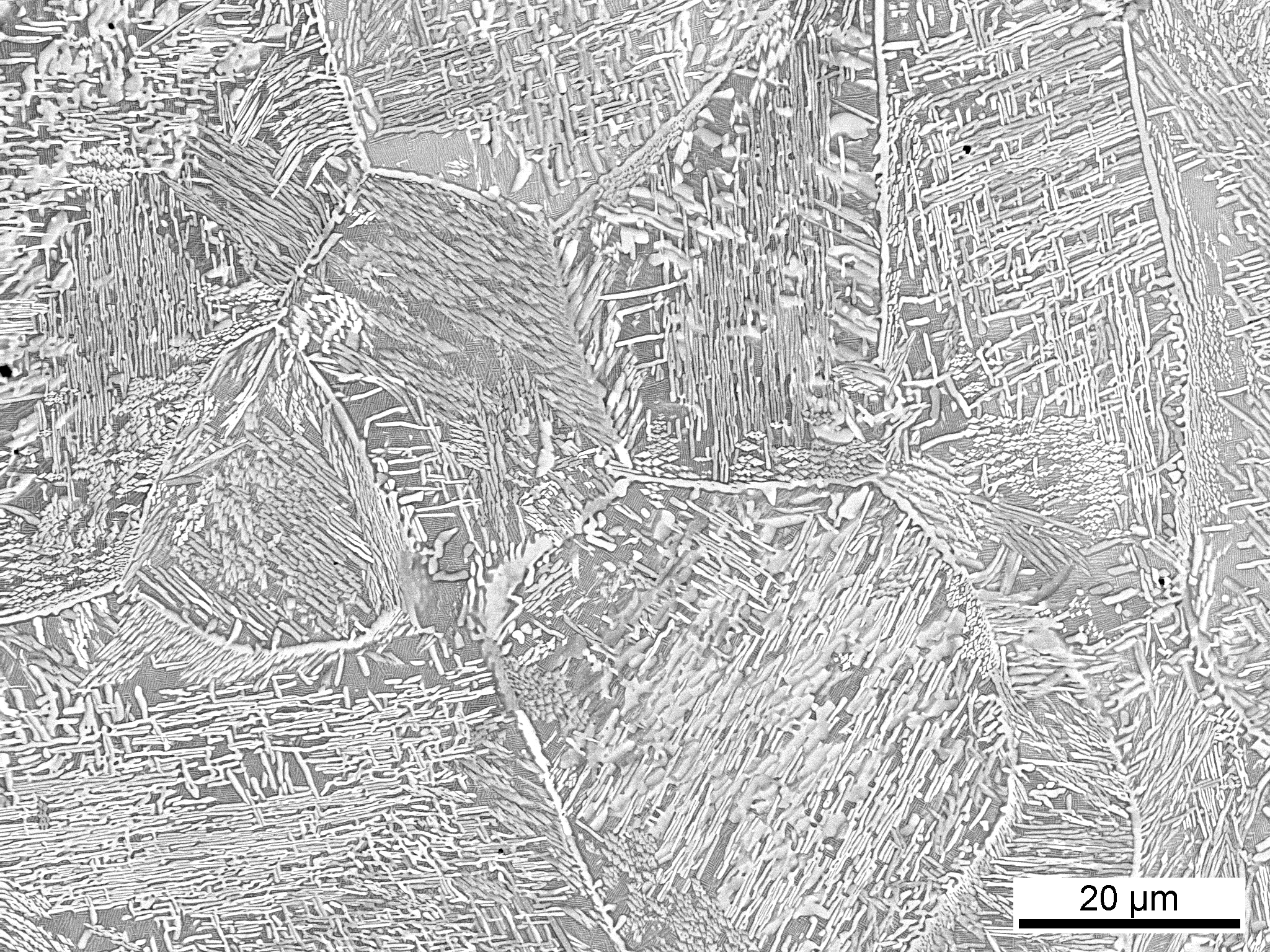

Supplement: Multimedia component 1 [file mmc1.zip › data in brief_supplementary material zip/BSE-Images_Microstructure evolution/Ti3/Ti3_As cast_1000x_.tif]

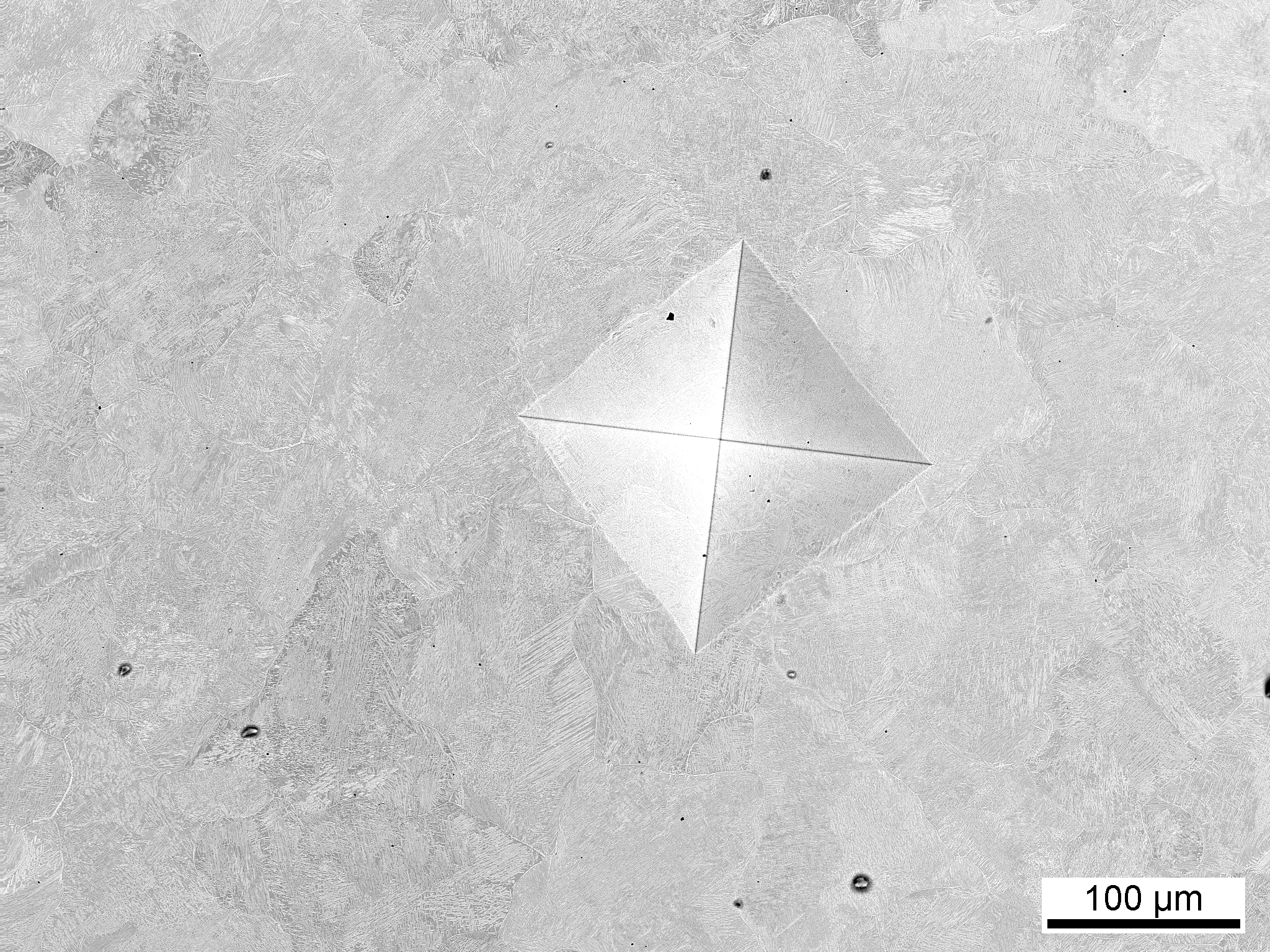

Supplement: Multimedia component 1 [file mmc1.zip › data in brief_supplementary material zip/BSE-Images_Microstructure evolution/Ti3/Ti3_As cast_174x_.tif]

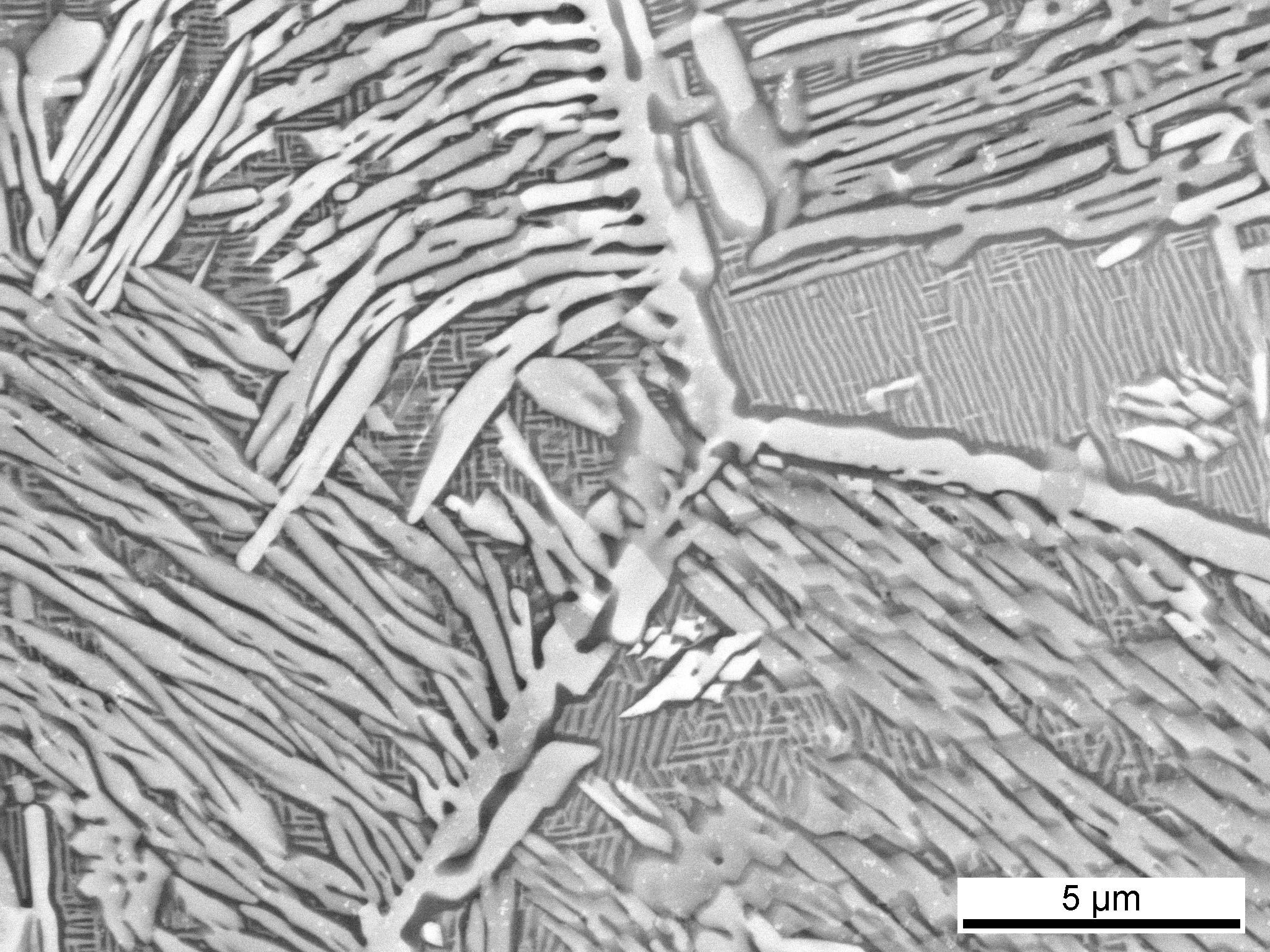

Supplement: Multimedia component 1 [file mmc1.zip › data in brief_supplementary material zip/BSE-Images_Microstructure evolution/Ti3/Ti3_As cast_5000x_.tif]

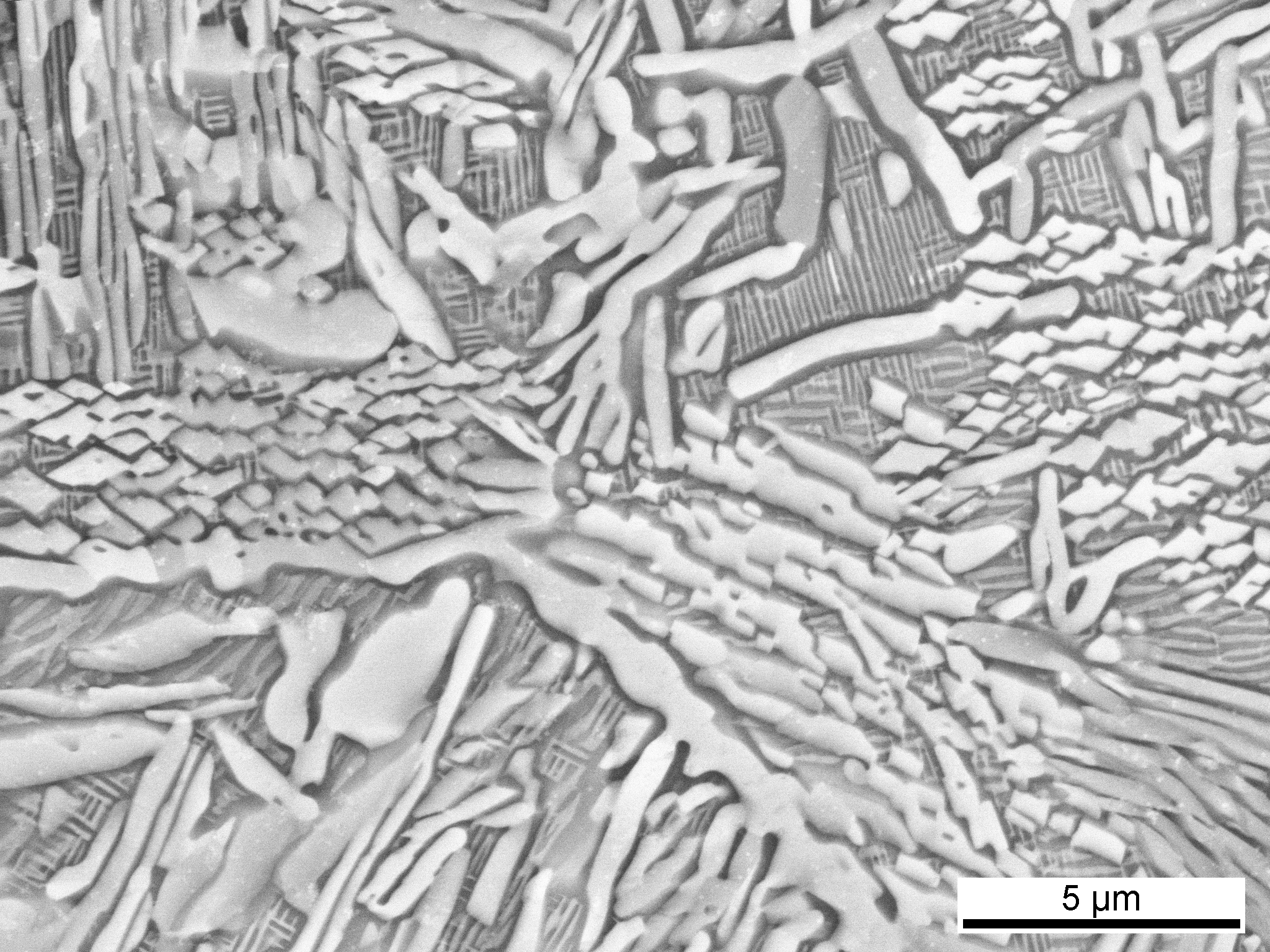

Supplement: Multimedia component 1 [file mmc1.zip › data in brief_supplementary material zip/BSE-Images_Microstructure evolution/Ti3/Ti3_As cast_5000x_.tiff]

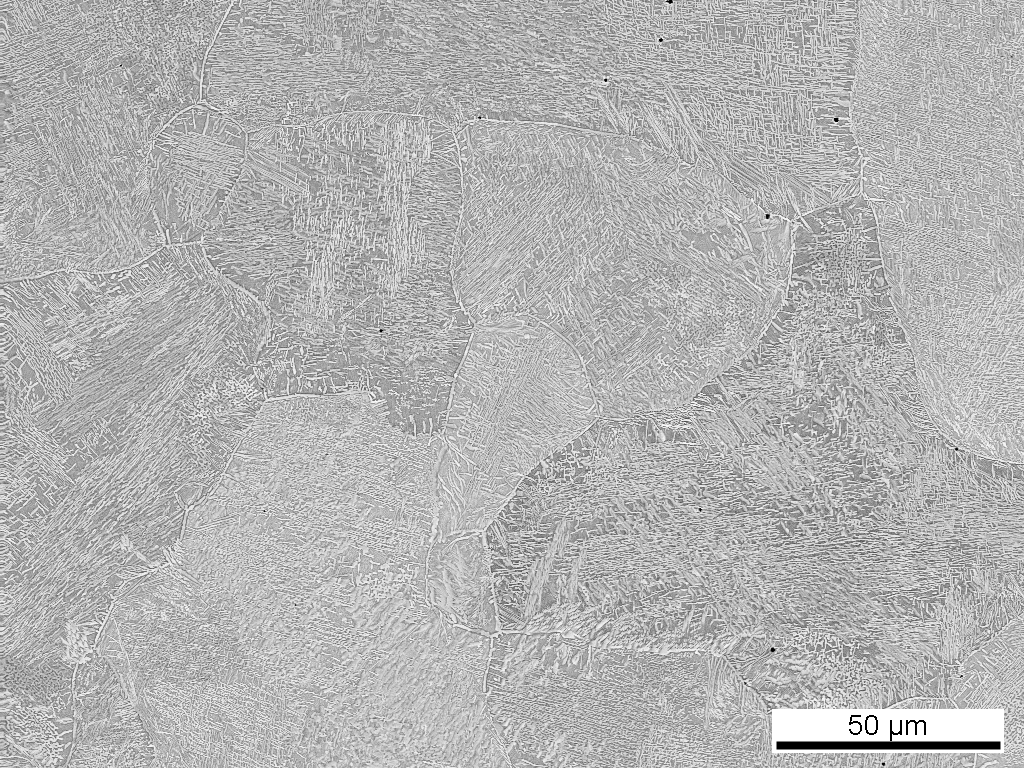

Supplement: Multimedia component 1 [file mmc1.zip › data in brief_supplementary material zip/BSE-Images_Microstructure evolution/Ti3/Ti3_As cast_500x_.tiff]

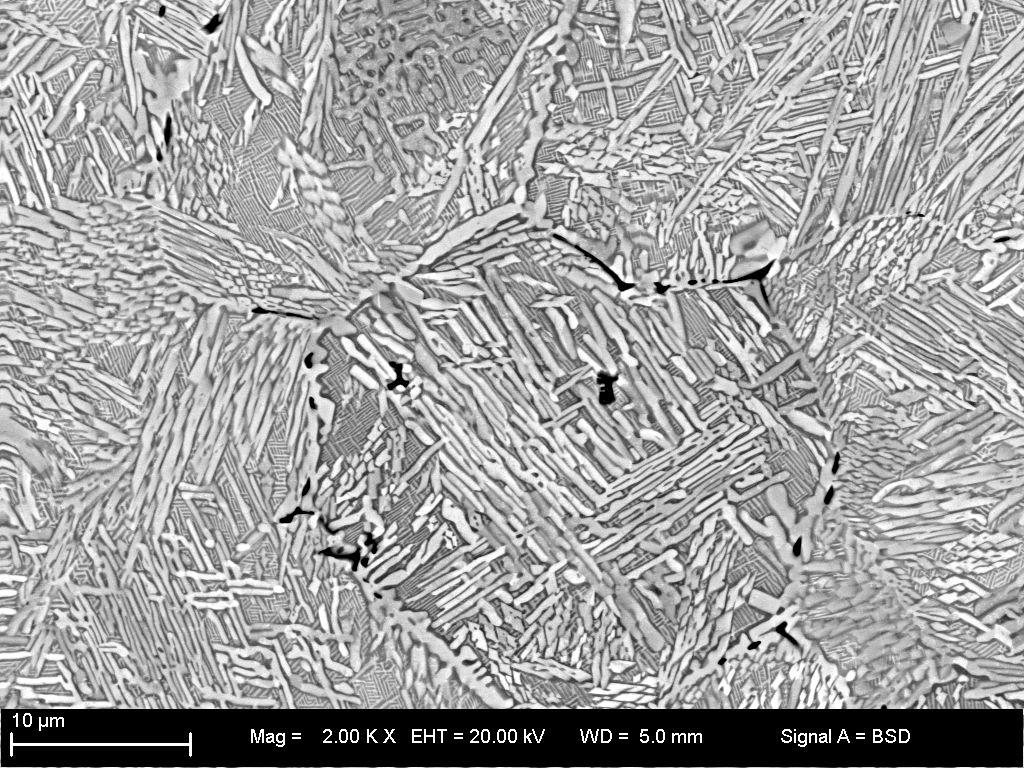

Supplement: Multimedia component 1 [file mmc1.zip › data in brief_supplementary material zip/BSE-Images_Microstructure evolution/Ti3C0.25/Ti3C0.25_As cast_2000x_.tif]

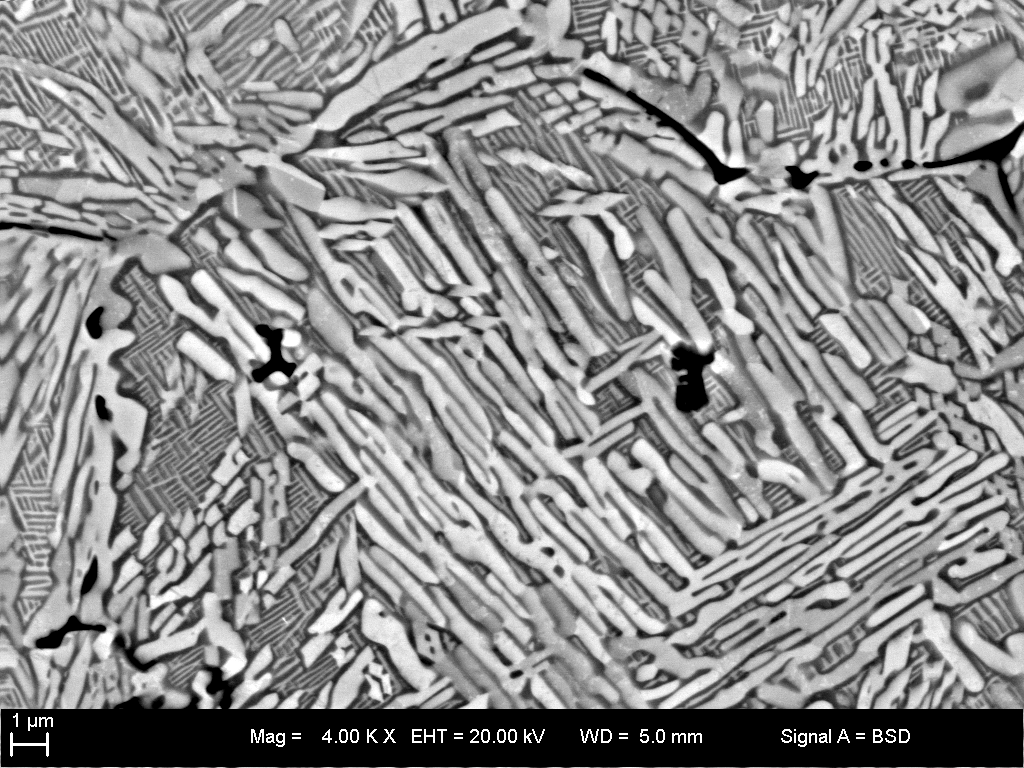

Supplement: Multimedia component 1 [file mmc1.zip › data in brief_supplementary material zip/BSE-Images_Microstructure evolution/Ti3C0.25/Ti3C0.25_As cast_4000x_ (2).tif]

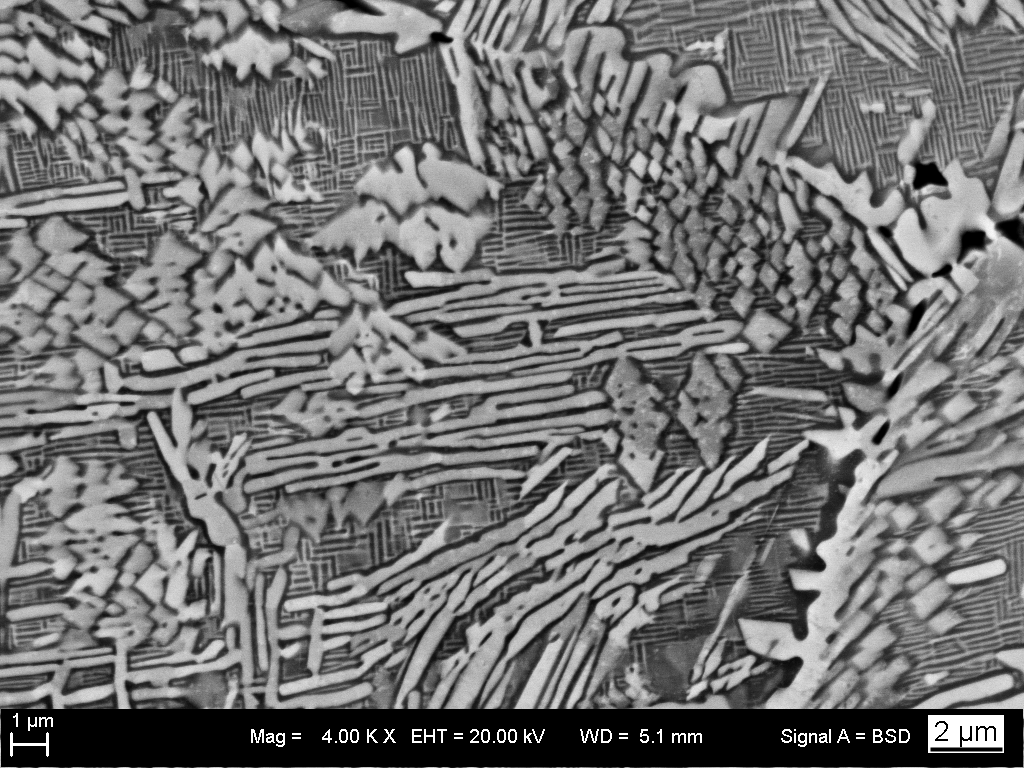

Supplement: Multimedia component 1 [file mmc1.zip › data in brief_supplementary material zip/BSE-Images_Microstructure evolution/Ti3C0.25/Ti3C0.25_As cast_4000x_.tif]

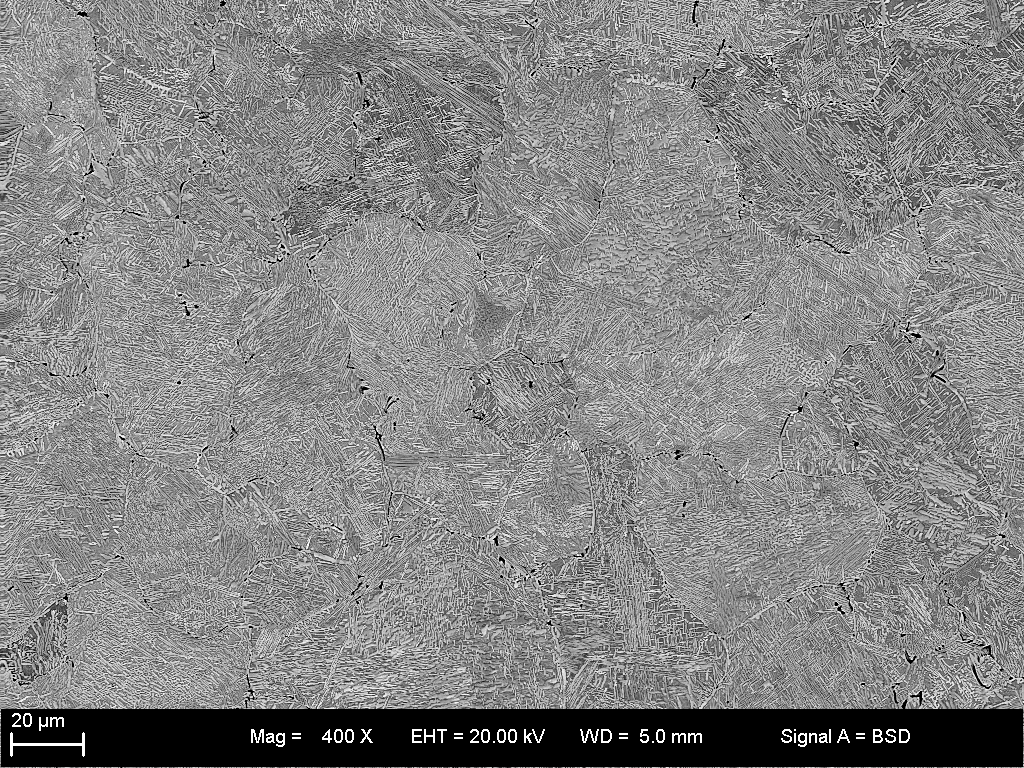

Supplement: Multimedia component 1 [file mmc1.zip › data in brief_supplementary material zip/BSE-Images_Microstructure evolution/Ti3C0.25/Ti3C0.25_As cast_400x_.tif]
